# Assessment of Trace Metals Concentration in Tree Barks as Indicator of Atmospheric Pollution within Ibadan City, South-West, Nigeria

**DOI:** 10.1155/2015/243601

**Published:** 2015-10-28

**Authors:** Ikechukwu P. Ejidike, Percy C. Onianwa

**Affiliations:** ^1^Department of Chemistry, Faculty of Science and Agriculture, University of Fort Hare, PB X1314, Alice 5700, South Africa; ^2^Department of Chemistry, Faculty of Science, University of Ibadan, Ibadan, Nigeria

## Abstract

Tree bark species were randomly collected from 65 sites having different anthropogenic activities, such as industrial, high traffic commercial, residential high and residential low traffic volume areas of Ibadan City, Nigeria. Levels of Cd, Cu, Pb, Zn, Co, and Cr of the dry-ashed bark samples were determined by AAS. The mean metal concentrations (mg kg^−1^) in samples from industrial zone were found as Pb: 3.67 ± 1.97, Cd: 0.10 ± 0.07, Zn: 30.96 ± 32.05, Cu: 7.29 ± 5.17, Co: 0.91 ± 0.58, and Cr: 2.61 ± 1.84. The trend of mean trace metal concentrations at high traffic commercial zone follows the order: Zn > Pb > Cu > Cr > Co > Cd. Residential high traffic and low traffic zones revealed the same trend as Cd < Co < Cr < Pb < Cu < Zn. Relatively strong positive correlation between the heavy metals at *ρ* < 0.05, such as Zn versus Cu (*r* = 0.79) and Co versus Cu (*r* = 0.77), was observed. The results of the study suggest that tree bark samples could potentially serve as bioindicators for Cu, Pb, Zn, Cr, and possibly Co and Cd. Furthermore, interspecies variation of heavy metal concentrations in plants barks is recommended.

## 1. Introduction

Air pollution was not perceived as a major problem in most countries until late 1960s and early 1970s; it was the global cooling forecast that captured public imagination in urban and industrialized areas [1]. In 2014, WHO released a report on air pollution, as the instigator of about 7 million people's death in 2012. These air contaminants are released into the ecosystem from immobile sources, such as power stations and other industries, and itinerant sources such as motor-powered vehicles, airplanes, and ships [2, 3]. The release of these gaseous volatile organic and inorganic pollutants in the form of particulate matters with sizes <10 × 10^−6 ^m [4] brings about steady deterioration of the air quality, giving rise to numerous cases of illness and even death, depletion of ozone layer, sulfurous and photochemical smog, acid rains, global warming, greenhouse effect, and physiological problem [3, 5]. One of the natural components (inorganic pollutants) of the environment is heavy metals, due to the proliferation of industries, high immigrant rate, and increased urbanization [6, 7]. Hence, they have been added to the ecosystem in large quantity, causing earth's atmosphere deterioration as it forms the life support of the planet [8, 9]. According to Hashmi et al. [10] heavy metals have been found to play an imperative role in the biochemical, biological, chemical, catabolic, metabolic, and enzymatic reactions in living cells [11] but when present in excess amount, they affect plants and animals leading to various kinds of diseases due to their stumpy levels in voluminous environmental and biological samples [12, 13].

Biological materials are used to ascertain atmospheric trace metal concentrations [14–16]. The use of biomonitor, as it is easy to collect and cheap, has higher concentrations than air and rainwater for measuring the trace metal concentrations in the atmosphere [17, 18]. Bioindicators of different entities are being used including vascular plants, mosses, woody plants, and lichens [19, 20]. The use of vegetal biomonitoring to evaluate air quality has been investigated; one alternative is to characterize gradients of air pollution on a small scale by the use of biological monitors [21, 22]. In San Luis Potosi, one of the most industrially developed cities in Mexico, Beramendi-Orosco et al. [23] studied the correlations between Zn, Pb, and Cu in tree-ring sequences of* Prosopis juliflora*, a tree species native to arid environments. The results suggested that the smelter's emissions are dispersed to longer distances through the tall chimneys, thereby confirming* Prosopis juliflora* as a good bioindicator, thus providing information on the chronology and sources of heavy metal pollution in urban and industrial areas. Turkish red pine (*Pinus brutia*), a widespread evergreen tree, has been investigated by Baslar et al. [14] in Mediterranean and Aegean regions of Turkey. The barks were examined as biomonitor of Zn and Mn accumulation in the studied area. Many studies have focused on the use of different tree bark species as biomonitor. However, in some studies only pine species were investigated to decide whether pine species can be used as a biomonitor for the determination of heavy metals. Results of such studies showed that the barks of the pine trees are good adsorbents of airborne pollutants, including anthropogenic heavy metals. Different authors have reported the atmospheric pollutants bioaccumulation of pine tree species, due to their widespread with evergreen needle normally 3–5 inches haphazardly warped [19, 22], including Turkish red pine (*Pinus brutia* Ten.) [14]. This study relates to a seasonal study in the city of Ibadan, with the aim to determine the levels of heavy metals using tree barks as indicators of atmospheric trace metal pollution. The metals investigated are Pb, Zn, Cd, Cu, Co, and Cr based on their environmental concerns.

## 2. Materials and Methods

### 2.1. Study Area

**Figure 1:**
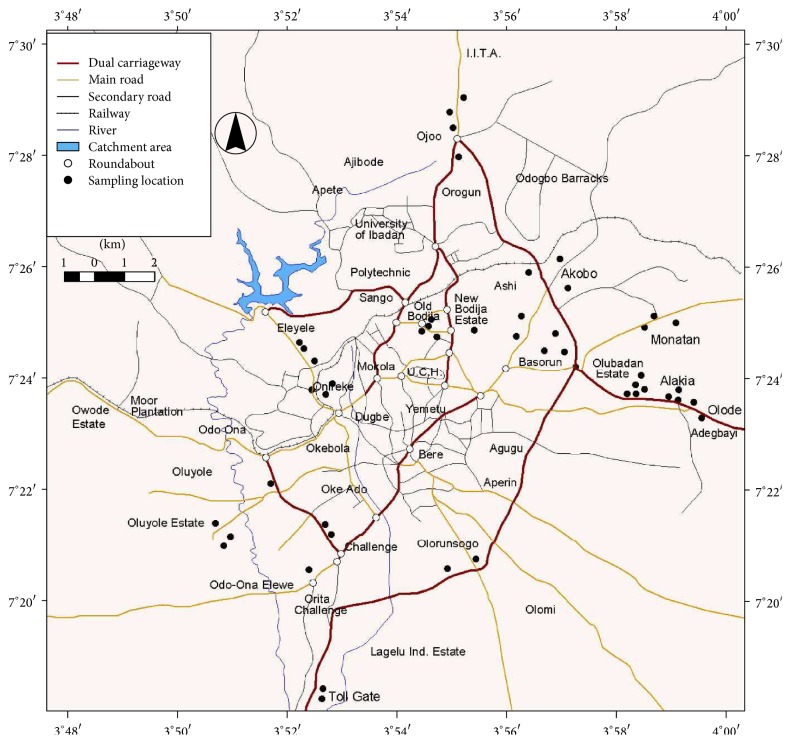
Ibadan City map showing the various sampling points.

Ibadan, located in southwestern Nigeria, is an ancient city categorized by urban sprawl and modernization [24]; it falls within the basement complex of the geological setting of southern Nigeria which lies between latitude 7°15′N–7°30′ and longitude 3°45′–4°60′E which possesses two seasonal climates (wet and dry) and moderately the same temperatures in a given season of the year. There has been increased industrialization and urbanization in Ibadan, leading to progressively increased discharges of heavy metals into the atmosphere [24]. The samples of tree backs with different bark morphology were collected from 65 locations within Ibadan city (Figure 1) involving seven different species, namely,* Terminalia catappa*,* Azadirachta indica, Gmelina arborea, Mangifera indica*,* Gliricidia sepium*,* Prosopis juliflora*, and* Murraya* species. Decision on sampling zones was made after a careful survey of the traffic densities, industrial distribution pattern covering the whole city of Ibadan. The sampling points designated and number of samples collected include industrial zone (INZ_1–20_), high traffic commercial zone (HTZ_1–14_), residential high traffic zone (RHZ_1–16_), and residential low traffic zone (RLZ_1–11_). The university botanical garden (CTZ_1–5_) aided in the uncontaminated reference point (control) being far-flung from traffic flow. The trees that were closer to the highways (about 5 m), about same age, were chosen and the barks were cautiously detached with a clean stainless knife at a height of about 1.8–2.0 m above the ground level [25, 26]. The samples were collected in duplicates and thereafter mixed to obtain whole sample.

### 2.2. Experiment

The samples were dried in the oven at a temperature of 60°C for 3-4 hours to a constant weight. The dried samples were pulverized to uniform size with a laboratory mill, thoroughly cleaned, and dried after each grinding to circumvent cross contamination. Samples were kept in clean plastic bags until the time analysis. About 2 g of the powdered sample was weighed accurately into a precleaned, numbered Vitreosil crucible. They were then transported into the muffle furnace and were preashed in the furnace for about 15 minutes at 150°C until the fumes vanished and ashed at the temperature of about 450–500°C [27]. The cooled ashed samples were then liquefied with 10 mL of 10% HNO_3_ solution and transfer to precleaned, labelled centrifuge tube. The crucible was again rinsed with portions of acid solution to make a total volume of 20 mL and vortexed for proper mixing. The samples were centrifuged for 30 minutes at 3000 rpm using HERMLE-Z323 Model. The supernatants were decanted into 100 mL calibrated volumetric flasks, made up to the mark with the acid solution, and evaluated for metal concentrations with the Buck Scientific Model 210 VGP Atomic Absorption Spectrophotometer possessing air-acetylene flame (functioned in line with the instrument's guidebook) and calibrated using mixed calibration standard solutions prepared as mandated [14, 28]. Blank solutions were prepared without the samples using the same procedure.

A spike recovery was used to verify the acid digestion procedure used in this study by spiking portions of previously analyzed samples, six samples including one with a high background or low background. These samples were oven-dried at 105°C, homogenized, and passed through the extraction and analytical steps [29]. The accuracy and validation of method were determined by analyzing spiked samples using the same reagents, apparatus, and method as those used for the samples. Percentage recovery of Cr, 94.20 ± 8.40%; Zinc, 97.10 ± 8.91%; Pb, 93.58 ± 15.87%; Cu, 94.93 ± 11.50%; Co, 88.40 ± 4.67%; and Cd, 97.32 ± 10.29% was obtained. The final concentrations of the metals measured in the tree bark samples by A.A.S technique are likely to be the corresponding percentages as the true values, since 100 ± 20% recovery is acceptable and all the metal concentrations were within the recovery range value [29–31]. The format used for the calculation of % recovery is as follows: Percent recovery (% Recovery) = (*A* − *B*)/*C* × 100%.

*A* is concentration of the spiked sample, *B* is concentration of the unspiked sample, and *C* is spike added.

## 3. Result and Discussion

**Figure 2:**
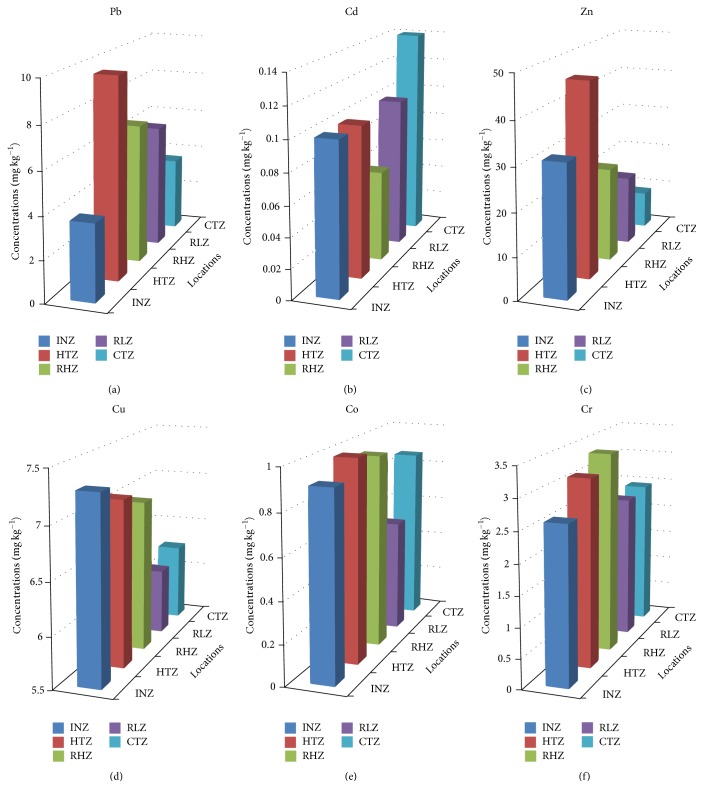
Metal concentrations in tree backs collected from all sampling zones. INZ: industrial zone; HTZ: high traffic commercial zone; RHZ: residential high traffic zone; RLZ: residential low traffic zone; CTZ: control zone.

The extractable heavy metal concentrations in the seven species of tree barks sampled from four zones (industrial, high traffic commercial and residential high and low traffic zones) in addition to the control samples (botanical garden, University of Ibadan) in Ibadan are presented in Table 1. Various heavy metals accumulation in different plant parts is dependent on the amount of metals present in the ecosystem, and the metal accumulation levels differ within and between species of plants [9, 32]. Generally, the same trend for metal concentration as found in the control zone was also observed in the industrial zone, residential high traffic zone, and residential low traffic zone that is Zn > Pb > Cu > Cr > Co > Cd. However, Zn showed the highest metal concentration with mean magnitude of 45.97 ± 43.88 mg kg^−1^ among all zones (Figures 2(a)–2(f)). Overall mean concentrations (mg kg^−1^, dry weight) of metals in tree bark samples obtained from this study were found to be Pb, 5.99 ± 5.25; Cd, 0.09 ± 0.07; Zn, 27.64 ± 29.66; Cu, 6.90 ± 5.54; Co, 0.87 ± 0.45; and Cr, 2.84 ± 1.58 which varied from 0.75 to 29.74, <0.01 to 0.26, 3.70 to 166.13, 1.81 to 39.12, 0.23 to 2.35, and <0.01 to 8.34 mg kg^−1^, respectively (Table 2). Abundance of metal concentrations follows the order: Zn > Cu > Pb > Cr > Co > Cd. The results indicate the presence of zinc, copper, and lead in high concentrations in samples taken from industrial, high traffic commercial and residential high traffic areas relative to residential low traffic and control areas. The concentrations of the heavy metal investigated in this study when compared with metal levels reported by Mleczek et al. [33] revealed that the concentrations obtained in this study were higher for metals like Pb, Zn, Cu, and Cr while the concentrations of Co and Cd were lower than those reported in another study; this could be due to the addition of these metals in trace amount in Nigeria petrol as a means of enhancing the octane number of gasoline [34, 35].

The results for the metal concentrations showed a marked variation for samples obtained from the industrial zone, Opposite Procter & Gamble Limited (INZ_5_); high traffic commercial zone, Iwo Roundabout (HTZ_13_); residential high traffic zone, Opposite Coca-Cola Mini Depot (RHZ_1_); and residential low traffic zone, Adeyi Avenue, Old Bodija (RLZ_3_); this generally has the highest value of metal levels. Zinc concentration varied widely in all zones with the residential low traffic zone having the lowest level 16.41 mg kg^−1^ and the highly trafficked commercial zone possessing the highest level with mean value of 45.97 mg kg^−1^. The highest concentration of Zn (166.16 mg kg^−1^) was observed in the sample collected at Iwo Road Roundabout (Alakia axis) (HTZ_14_) within high traffic commercial zone. The results showed that there is a significant difference in the zinc concentration at *ρ* < 0.05 among the zones. However, no significant difference was observed in the zinc concentration of high traffic commercial zone as compared to industrial zone. This indication connotes emission and tyre wear from motor vehicle pointers as a source of the environmental zinc contamination. Also, zinc could be added to the environment during industrial activities like engine wear, waste combustion, exhaust emission, and the use of sewage sludge from industrial areas as fertilizer. Zn promotes growth and development in the human body but its excessiveness may be an indication of metal poisoning and growth impedance [36].

The concentration of lead in samples obtained from high traffic commercial, residential high traffic, and residential low traffic zones varied slightly with average concentrations of 9.62, 6.70, and 5.96 mg kg^−1^, respectively. However, possible cradles of metal contaminants in the environment are a crucial part of environmental pollution studies. The chief source of lead (Pb) is the use of leaded gasoline in the 20th century [37–40], which inevitably brings about lead pollutant in the ecosystem. Lead content of Nigeria petrol [0.6–0.8 g L^−1^] ranks among the world highest content [41]. Concentrations of lead reported in other studies showed higher values than that obtained in this study except for lead concentration reported by [29, 33, 42, 43]. Human exposure to lead brings about reproductive dysfunction that exhibits biochemical-morphological features including decreased sperm quality, disorganized epithelia, and altered sperm morphology [44]. The analysis of variance showed no significant difference between the levels of copper in all zones at *ρ* > 0.05. Copper as a crucial micronutrient is essential for bone growth and formation, neurologic systems, and nervous systems myelin sheaths [45]. The high level (39.12 mg kg^−1^) of copper concentration in samples sampled from Aworawo along Ojoo road (HTZ_9_), high traffic commercial zones, could result from various human activities. Close look at the results of copper level in residential high and low traffic zones suggests common source. Increased levels could lead to cirrhosis, rheumatoid arthritis, malnutrition, irregular hair growth and depigmentation, fur, growth impairment and reproductive performance, failure of the heart, and disturbances in gastrointestinal systems [44].

Mean concentration of cobalt in the zones was found to follow the trend: high traffic commercial > residential high traffic > industrial > control > residential low traffic (Figures 2(a)–2(f)), with ranges. There is no significant difference observed for the level of cobalt in all investigated zones at *ρ* > 0.05. The highest cobalt concentration was found in the* Mangifera indica* sample from behind Sumal Foods (chewing gum) (INZ_3_), industrial zone. Chromium levels were largely low in the study (Figures 2(a)–2(f)), and the residential low traffic zone posed the lowest level of chromium, which could be attributed to natural sources. In various zones, chromium pollution could be due to automobile engine and body erosion, yellow lead chromate paint for road pattern, and few steel and glass industry activities [46–48]. Chromium is known as a ubiquitous pollutant from the industrial and environmental activities, also a known human carcinogen, with specificity in cancer of the lungs [49, 50]. There is no definite gradient in the concentration of cadmium present in the investigated zones. The residential low traffic zone gave 0.10 mg kg^−1^ and the lowest mean level of cadmium (Cd) was found in residential high traffic zone (0.06 mg kg^−1^) ranging from 0.01 to 0.15 mg kg^−1^. The slight increase in the mean level of cadmium in the control zone (0.14 mg kg^−1^), with the highest concentration found in the* Gmelina arborea* species (0.25 mg kg^−1^), could be linked to the use of phosphate fertilizer which has cadmium as a trace element for growing crops. When cadmium is accumulated above the recommended threshold limit, it brings about liver acute and chronic poisoning and also the replacement of calcium within bone matrix of young children [44]; it inhibits increased oxidative stress resulting in the damage of membrane and membrane-enzymes bound losses [51].

### 3.1. Correlation Matrix

A coefficient matrix among Pb, Cd, Zn, Cu, Co, and Cr concentrations in samples from the studied locations are presented in Table 3. The results showed that the elements investigated in this study are highly correlated with one another, suggesting common source and other possible uncommon sources of metal fallout. There is relatively high correlations amongst metals such as Zn and Cu and Co and Cu. Positive moderate correlation coefficient was observed between Pb and Zn and Zn and Co. The high correlation found between Pb and Zn and Zn and Cu suggests a common source and other possible well-mixed constituents initiating from different sources. Positive moderate correlation coefficient was also observed between Cr versus Pb and Zn and Cr versus Cu and Co. Zn level in all the zones was seen to be in high concentration followed by Cu and Pb. High values of Cu and Zn obtained indicate that emission and tyre wear from motor vehicle as a source of the environmental zinc contamination; high level of copper could come from the fabrication of brass alloy, brake linings, and electrical and mechanical working while lead could be the use of leaded gasoline in the Nigeria petrol. This is because lead additives in the form of tetraethyl lead (TEL[Pb(C_2_H_5_)_4_]) are added to gasoline as the cheapest means of boasting the octane number of Nigeria petrol and zinc in the form of organometallic compounds such as zinc dialkyl dithiophosphate (ZDDP), lubricating oils, and tyres [46, 52, 53].

## 4. Conclusion

The results of this survey demonstrate the suitability of* Terminalia catappa*,* Azadirachta indica*,* Gmelina arborea*,* Mangifera indica*,* Prosopis juliflora*,* and Murraya* species, and* Gliricidia sepium* bark species as potential bioindicators for Cu, Pb, Zn, Cr, and possibly Co and Cd as well as the pointers of some degree of temporal heavy metals contamination. The levels of the metals were higher in bark samples from highly trafficked commercial zone than in other zones. The deviation in metal concentrations amongst the studied sites could be due to various anthropogenic activities. With respect to the high contribution factors of Zn, Cu, and Pb to the potential ecological risk of the area, thorough study should be piloted to determine the actual route of the metals entry to the environment. It is imperative that the environmental trace metal levels be constantly examined to diagnose the state and trend of environmental atmospheric pollution. Further monitoring of plant barks species as well as the surrounding lower epiphytes, higher epiphytes, and vascular plants is needed in order to determine whether any temporal trends exist in metal concentration among biological indicators. Potential risk assessment of these heavy metals to crops, animals, and humans in the area is recommended. Further, farming activities within the diameter of 15–10 Km of the vicinity of the industries and high human activities should be discouraged as there will be every possibility of heavy metals uptake by planted crops, which will in turn reach up the food chain and finally to humans. Furthermore, interspecies variation of heavy metal concentrations in plants barks is also recommended.

## Figures and Tables

**Table 1:** Summary of results for tree bark metal concentration (mean ± SD, mg kg^−1^, dry weight) in all zones.

Metal	Parameter	Industrial zone	High traffic commercial zone	Residential high traffic zone	Residential low traffic zone	Control zone
Pb	Mean ± SD	3.67 ± 1.97	9.62 ± 7.52	6.70 ± 3.52	5.96 ± 6.99	3.59 ± 2.08
Cd	Mean ± SD	0.10 ± 0.07	0.10 ± 0.06	0.06 ± 0.05	0.10 ± 0.07	0.14 ± 0.07
Zn	Mean ± SD	30.96 ± 32.05	45.97 ± 43.88	22.24 ± 12.93	16.41 ± 16.73	8.75 ± 5.39
Cu	Mean ± SD	7.29 ± 5.17	7.11 ± 9.77	6.97 ± 3.78	6.14 ± 2.43	6.25 ± 2.42
Co	Mean ± SD	0.91 ± 0.58	0.99 ± 0.49	0.95 ± 0.27	0.55 ± 0.26	0.86 ± 0.33
Cr	Mean ± SD	2.61 ± 1.84	3.12 ± 1.61	3.35 ± 1.16	2.39 ± 1.82	2.45 ± 0.50

**Table 2:** Overall mean metal concentrations (mg kg^−1^, dry weight) in tree bark samples.

Parameters	Pb	Cd	Zn	Cu	Co	Cr
Total	389.06	5.99	1796.89	448.47	56.64	184.81
Mean ± SD	5.99 ± 5.25	0.09 ± 0.07	27.64 ± 29.66	6.90 ± 5.54	0.87 ± 0.45	2.84 ± 1.58
Range	75–29.74	<0.01–0.26	3.70–166.13	1.81–39.12	0.23–2.35	<0.01–8.34

**Table 3:** Correlation coefficient among metals in tree bark samples from all zones (bold correlations are significant at *ρ* < 0.05).

	Pb	Cd	Zn	Cu	Co	Cr
Pb	1					
Cd	**0.40**	1				
Zn	**0.70**	0.31	1			
Cu	0.27	**0.50**	**0.79**	1		
Co	0.24	0.20	**0.54**	**0.77**	1	
Cr	**0.67**	0.24	**0.55**	**0.63**	**0.69**	1
